# Postoperative muscle atrophy and fatty degeneration with respect to surgical approaches in total hip arthroplasty

**DOI:** 10.1007/s00402-025-05793-0

**Published:** 2025-03-08

**Authors:** Grigorios Svarnas, Vlad Popa, Theofania-Sotiria Patsiou, Joseph Michael Schwab, Moritz Tannast

**Affiliations:** 1 Department of Orthopedic Surgery and Traumatology, Fribourg Cantonal Hospital (HFR), University of Fribourg, Fribourg, Switzerland; 2 Faculty of Science and Medicine, University of Fribourg, Fribourg, Switzerland; 3 Department of Biology, University of Bern, Bern, Switzerland; 4 Insel Department of Orthopaedic Surgery and Traumatology, Inselspital, Bern University Hospital, University of Bern, Bern, Switzerland

**Keywords:** Anterior hip approach, Anterolateral hip approach, Transgluteal hip approach, Posterior hip approach, Cross-sectional area, Goutallier

## Abstract

**Background:**

Total hip arthroplasty is the gold standard for treatment of hip osteoarthritis. The different surgical approaches utilize different intervals to access the hip joint. There is concern that some surgical approaches cause soft tissue trauma resulting in post-operative muscle weakness of patients undergoing THA. We therefore asked whether the implantation of a total hip prosthesis by each of four common surgical approaches (anterior, anterolateral, direct lateral and posterior) leads to (i) muscle atrophy (defined as decreased muscle cross-sectional area [CSA]) and (ii) muscle degeneration (defined as fatty infiltration) of 12 specific periarticular hip muscles. Further, if significant change is found, can we establish an associated pattern with a particular surgical approach?

**Method:**

We retrospectively evaluated 493 patients undergoing computed tomography of the pelvis in HFR hospital Fribourg, Switzerland, between 2014 and 2020. All patients had undergone a primary THA at some point prior to their CT scan. Trauma, metastasis, bone tumor, neurologic disorder, infection, and revision cases were excluded. Twelve periarticular hip muscles were measured for CSA and degree of fatty infiltration according to the Goutallier scale on axial and sagittal views of both the operative and nonoperative hips.

**Results:**

CSA of the operative hip muscles differed significantly depending on approach. Similarly, there was a statistically significant difference in muscle degeneration in the operative hips according to the Goutallier classification. We observed a specific level and pattern of muscle atrophy for each approach.

**Conclusion:**

In all approaches, there is a trade-off between the muscles they affect, their role, and whether there is a possibility of partial compensation by other muscles. The anterior approach was the least harmful to the gluteus medius muscle.

## Introduction

Total hip arthroplasty (THA) for the treatment of hip osteoarthritis is one of the most successful orthopaedic surgical procedures [1–3]. According to the Swiss National Hip & Knee Joint Registry (SIRIS) 2023 Annual Report [4] the four most common approaches used during primary THA in Switzerland from 2017–2022 were: anterior approach (52.3% of all primary THAs) [5–7], the anterolateral approach (30.2% of all primary THAs) [8], the posterior approach (13.0% of all primary THAs) [9], and the transgluteal or direct lateral approach (4% of all primary THAs) [10–12].

**Fig. 1:**
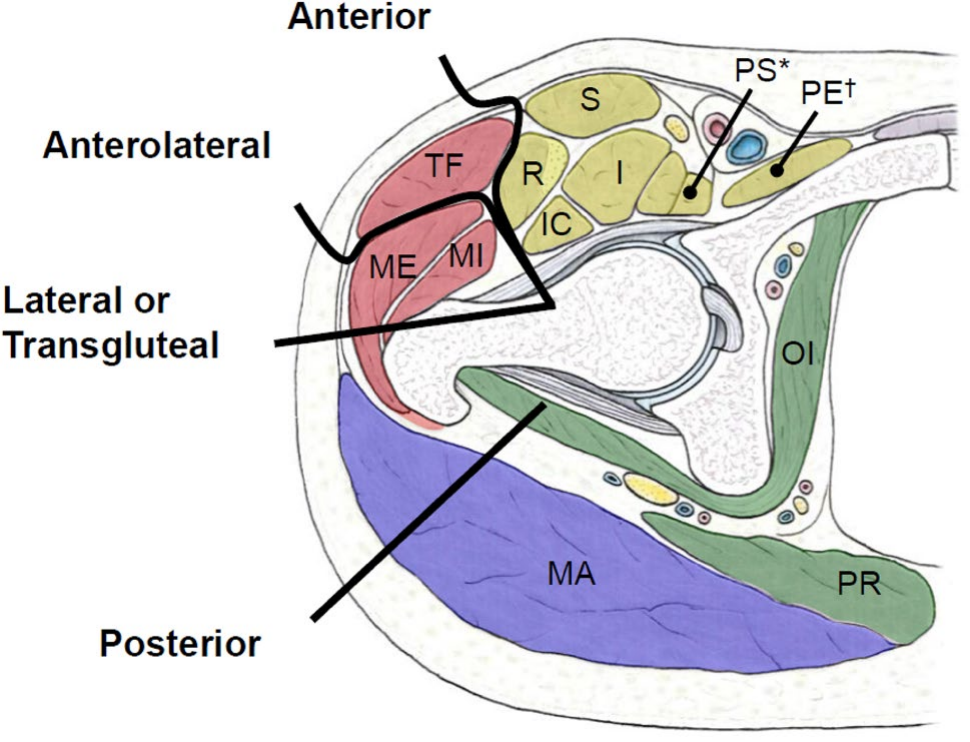
Intervals by total hip arthroplasty surgical approach. The anterior approach accesses the hip through the interval between the tensor fascia lata and sartorius muscles and does not necessitate detachment of the tendinous insertions or retraction of muscle. The anterolateral approach develops the interval between the tensor fascia lata and gluteus medius muscles. The direct lateral approach requires elevation of the hip abductors (gluteus medius and gluteus minimus muscles) to access the joint. The posterior approach accesses the joint by detaching the piriformis and short external rotator muscles from the femur, thus providing access to the posterior joint capsule. The colors indicate groups of muscles with the same innervation (yellow = femoral nerve, red = superior gluteal nerve, blue = inferior gluteal nerve, green = separate innervation) *Anatomical features shown: TF: tensor fascia lata; ME: gluteus medius; MI: gluteus minimus; R: rectus femoris; S: sartorius; I: iliacus; PS: psoas; PE: pectineus; OI: obturator internus; PR: piriformis; MA: gluteus maximus*

Surgical approaches for THA can be categorized by how they navigate muscular (intramuscular or intermuscular) and/or nervous (intranervous, internervous) planes. (Fig. 1). Preserving periarticular muscles, especially during surgical intervention, is important to optimize postoperative hip function and health [13–15]. For example, tenotomy and retraction of the gluteus medius and minimus muscles during the direct lateral approach is associated with postoperative gait abnormalities and abductor dysfunction [16]. Likewise, failed repair of the short external rotators during a posterior approach is reported to be one contributing factor to postoperative hip instability [17, 18]. Intranervous approaches, while trying to avoid direct muscle injury, can be an additional cause of muscle degeneration [19].

While modern “minimally invasive” surgical approaches emphasize tissue preservation, muscle damage has been detected in these approaches [20–22]. The current literature on muscle atrophy and degeneration following THA is heterogeneous and incomplete. Limitations of existing studies are that they only assess two approaches, involve a small cohort of patients [23–25], assess an incomplete subset of the periarticular hip muscles [26], do not assess fatty muscle degeneration, or limit their assessment of muscle damage to cadavers [20]. This observation has motivated us to quantify muscle damage in a large series of total hip arthroplasties for the four most common surgical approaches for primary THA in our region.

We therefore asked: what is (1) the cross-sectional area and (2) degree of fatty infiltration on computed tomography for a comprehensive set of periarticular hip muscles after primary THA by the four most common THA approaches used in our region? In addition, we asked how these measurements compare to the same measurements performed on the same set of periarticular hip muscles on the nonoperative side?

## Materials and methods

**Fig. 2:**
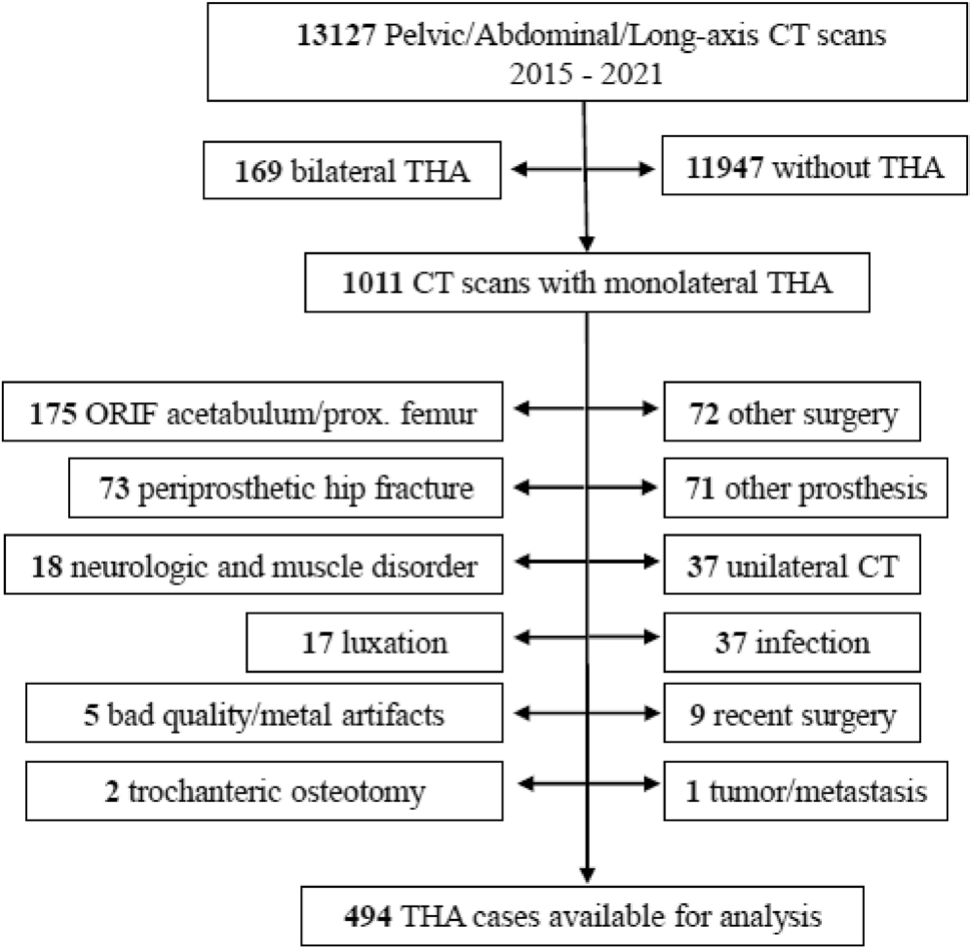
Exclusion criteria for the study sample

We conducted a retrospective comparative study on patients who had undergone primary THA using one of four surgical approaches. This study was approved by the appropriate governing Institutional Review Board (IRB) (CER-VD 2021–00577). We included all 13,127 patients undergoing computed tomography (CT) of the pelvis at the Fribourg Cantonal Hospital, Switzerland, between 2015 and 2021 for any reason. We excluded all patients without THA, bilateral THA patients, or those with inadequate imaging such as unilateral CT (scan not covering both sides) or a scan lacking metal artifact suppression. Patients were also excluded if their medical records or imaging indicated that their CT scan was completed within the first 12 months following THA. Additionally, patients were excluded if, at the time of their CT, they were being treated for tumor/metastasis, infection, had known neurological or muscle disorders, previous surgery (other than THA), periprosthetic fractures, dislocation after hip arthroplasty or trochanteric osteotomy for complex reconstructions (Fig. 2). We also excluded patients if there was inadequate documentation regarding the surgical approach used during their primary THA. This left us with 493 cases available for analysis.

### Assessment of muscular atrophy and fatty infiltration

Our approach was based on a modified protocol by Glynn et al*.* [27] where we used five axial cuts and one sagittal cut from the pelvis CT scans to assess 12 specific periarticular hip muscles (Table 1; Figure A.1). This method has been shown reliable and reproducible. Atrophy of each muscle was assessed by measuring the cross-sectional area (CSA; cm^2^). Fatty infiltration of each muscle was assessed using the Goutalier classification [28, 29]. Two of the authors (GS and VP) performed all measurements using Centricity Universal Viewer (Version 6.0 GE HealthCare, USA).

**Table 1:** Muscle cross-sectional area and fatty infiltration of the 12 hip muscles were assessed at five levels on axial CT-scans views and one level at the sagittal view

Muscle	Plane	Level/position	Description of CSA measurement
Gluteus maximus	Axial	level of the greater trochanter tip	Maximum CSA
Gluteus medius	Axial	level of the greater trochanter tip	Maximum CSA
Gluteus minimus	Axial	level of the acetabular roof	Maximum CSA
Iliacus	Axial	level of the greater trochanter tip	Maximum CSA
Psoas	Axial	level of the greater trochanter tip	Maximum CSA
Oturator internus	Axial	level of the “tear drop”	Maximum CSA, including tendinous portion
Obturator externus	Sagittal	through the transverse acetabular ligament	Maximum CSA
Piriformis	Axial	level of the sciatic notch	Maximum CSA
Rectus femoris, direct head	Axial	level of ischial tuberosity	Maximum CSA
Tensor fascia latae	Axial	level of the greater trochanter tip	Maximum CSA
Sartorius	Axial	level of the greater trochanter tip	Maximum CSA
Vastus lateralis	Axial	level of ischial tuberosity	Maximum CSA

### Study subgroups

All 493 cases were grouped based on surgical approach: anterior (Hueter approach; [5, 7]), anterolateral (Watson-Jones/OCM/Röttinger approach; [8, 10, 11]), posterior (Moore approach [9]), and direct lateral (Bauer/Hardinge approach [10, 11]). The surgical approach was determined from one or more of the following sources: hospital patient charts, operative reports, direct communication with the surgeon, and/or the Swiss Implant Joint Registry (SIRIS).

### Demography

All patients were evaluated for age, sex, side, height, weight, and body-mass-index, which did not differ significantly among the four study subgroups (Table 2). Overall, no significant difference was found in the CSA (but see sartorius; p < 0.05) and Goutallier fatty infiltration classification (but see gluteus minimus; p < 0.05) of the contralateral (nonoperative) hip muscles between the four groups (Table 3) [28, 29].

**Table 2:** Demographics for each group

Parameter	Anterior	Anterolateral	Direct lateral	Posterior	p-value
Number of hips	145	38	203	107	
Age at surgery [years]	70 ± 10 (42–88)	69 ± 10 (43–82)	67 ± 10 (34–93)	65 ± 12 (26–86)	0.029
Side [% right]	84 (57.93%)	17 (44.73%)	105 (51.72%)	62 (57.94%)	0.32
Sex [% female]	61 (42.36%)	14 (36.68%)	83 (40.88%)	50 (46.72%)	0.68
Height [m]	1.69 ± 0.11 (0.82–1.94)	1.69 ± 0.1 (1.5–1.85)	1.69 ± 0.18 (1.45–1.9)	1.69 ± 0.21 (1.5–1.86)	0.34
Weight [kg]	80 ± 19 (52–166)	79 ± 17 (57–140)	80 ± 20 (45–173)	80 ± 20 (42–170)	0.92
Body mass index [kgm^−2^]	29.41 ± 21.66 (17.26–46.81)	26.42 ± 3.89 (15–31.24)	28.31 ± 6.14 (16.51–53.62)	28.31 ± 6.68 (16.45–52.46)	0.62

**Table 3:** Comparison of non-operative hip muscle quality (cross-sectional area, Goutallier grade) for the different study groups. The study groups were comparable

Muscle	Anterior	Anterolateral	Direct lateral	Posterior	p-value	
Gluteus maximus	43.89 ± 11.36 (20.83–78.54)	42.38 ± 13 (13.99–78.84)	41.65 ± 10.99 (17.78–79.06)	44.41 ± 12.92 (21.17–96.9)	0.197	Cross-sectional area contralateral side
Gluteus medius	10.4 ± 2.72 (4.09–17.61)	9.49 ± 2.68 (4.94–16.01)	9.9 ± 2.81 (4.27–20.64)	10.54 ± 3.3 (4.62–23.13)	0.103	
Gluteus minimus	10 ± 2.41 (5.59–18.97)	9.51 ± 2.48 (5.17–15.91)	9.56 ± 2.26 (4.95–19.66)	9.91 ± 2.47 (5.51–17.65)	0.409	
Iliacus	5.35 ± 1.53 (2.31–10.83)	5.19 ± 1.3 (2.21–7.46)	4.94 ± 1.46 (2.09–9.17)	5.13 ± 1.64 (2.02–9.74)	0.138	
Obturator internus	13.67 ± 3 (6.65–23.44)	12.89 ± 2.69 (8.79–20.68)	12.98 ± 2.95 (6.56–20.39)	12.85 ± 2.82 (7.03–22.19)	0.074	
Obturator externus	7.64 ± 1.76 (3.84–13–84)	8 ± 2.42 (3.44–14.98)	7.43 ± 1.83 (4.11–14.89)	7.48 ± 1.78 (3.56–14.22)	0.286	
Piriformis	11.1 ± 2.48 (5.33–18.78)	10.89 ± 2.4 (7.16–16.85)	10.97 ± 3.08 (3.31–19.75)	10.73 ± 3.25 (3.9–25.09)	0.641	
Psoas	1.5 ± 0.52 (0.6–3.02)	1.43 ± 0.48 (0.7–2.8)	1.4 ± 0.45 (0.58–2.72)	1.42 ± 0.5 (0.49–3.49)	0.491	
Rectus femoris	5.76 ± 1.82 (2.51–12.61)	5.95 ± 2.12 (3.08–11.66)	5.36 ± 1.6 (2.45–9.49)	5.59 ± 1.7 (2.07–9.54)	0.251	
Sartorius	3.07 ± 1.03 (1.06–7.79)	3.02 ± 0.86 (1.3–5.07)	2.77 ± 0.93 (0.93–5.74)	2.83 ± 0.92 (1.16–4.88)	0.028	
Tensor	6.07 ± 2.22 (2.6–12.59)	5.85 ± 1.41 (3.39–8.87)	5.58 ± 2.16 (1.73–12.92)	6.18 ± 2.89 (2.29–15.58)	0.167	
Vastus lateralis	5.24 ± 1.95 (1.09–12.69)	5.22 ± 1.9 (2.04–12.23)	4.83 ± 1.91 (1.19–14.33)	4.76 ± 1.62 (1.59–12.77)	0.063	
Gluteus maximus	0%	0%	0.5%	0%	0.698	Fatty degeneration of contralateral side in groups of slight (0–2) and severe (3–4) Goutallier grade
Gluteus medius	0.7%	0%	2.5%	5%	0.188	
Gluteus minimus	2.1%	2.6%	2.8%	8.4%	0.025	
Iliacus	0%	0%	0%	0%	N/A	
Obturator internus	0%	0%	0%	0%	N/A	
Obturator externus	0%	0%	0%	0%	N/A	
Piriformis	0%	0%	0%	0%	N/A	
Psoas	0.7%	0%	1%	0%	0.706	
Rectus femoris	0%	0%	0%	0.9%	0.305	
Sartorius	0%	0%	0.5%	0%	0.695	
Tensor	0%	0%	1%	0%	0.409	
Vastus lateralis	0%	0%	0%	0%	N/A	

### Data analyses

We used the Kolmogorov–Smirnov test to assess normal distribution. When comparing CSA and fatty infiltration scores of the nonoperative side as “baseline” values for each group, we applied an unpaired Kruskal–Wallis test for CSA [30], and a chi-square test for fatty infiltration [31]. CSA was compared between the operative and nonoperative sides within each subgroup using the Wilcoxon paired test [32]. Goutallier fatty infiltration classification was subdivided into “non-severe” (Goutallier classification grade 0–2) and “severe” (Goutallier classification grade 3–4) and the operative and nonoperative sides within each subgroup were compared using the chi-square test.

Additional analysis on our dataset to quantify differences per approach within muscles was performed by fitting a Marcov Chain Monte Carlo generalized linear mixed-effects model (MCMCglmm [33],). The full description of this analysis, as well as the results, are provided in the Appendix A.

## Results

### CSA postoperatively

Statistically significant differences in the CSA of multiple muscles were observed in all four surgical approaches when comparing the operated to the non-operated side (Table 4). For the anterior approach, we found a statistically significant decrease in CSA for the gluteus minimus (9.63 vs 10.0 mm^2^; p = 0.009), iliacus (5.02 vs 5.35 mm^2^; p < 0.001), obturator internus (12.02 vs 13.67 mm^2^; p < 0.001), piriformis (9.99 vs 11.1 mm^2^; p < 0.001), and psoas (1.38 vs 1.5 mm^2^; p = 0.01) muscles, whereas we found an increase of CSA for the rectus femoris (6.02 vs 5.76 mm^2^; p = 0.01) and sartorius (3.29 vs 3.07 mm^2^; p < 0.001) muscles. For the anterolateral approach, a statistically significant decrease in CSA was noted for the gluteus minimus (8.85 vs 9.51 mm^2^; p = 0.05), obturator internus (11.68 vs 12.89 mm^2^; p = 0.007), and tensor (4.96 vs 5.85 mm^2^; p < 0.05) muscles. For the posterior approach, a statistically significant decrease in CSA was noted in the gluteus minimus (9.28 vs 9.91 mm^2^; p = 0.001), iliacus (4.79 vs 5.13 mm^2^; p = 0.009), obturator internus (9.63 vs 12.85 mm^2^; p < 0.001), piriformis (8.82 vs 10.73 mm^2^; p < 0.001), and tensor (5.6 vs 6.18 mm^2^; p = 0.01) muscles; a significant increase in CSA was found for the sartorius (3.0 vs 2.83 mm^2^; p < 0.001) muscle. For the direct lateral approach, a statistically significant decrease in CSA was noted for the gluteus medius (9.52 vs 9.9 mm^2^; p = 0.013), gluteus minimus (8.05 vs 9.56 mm^2^; p < 0.001), iliacus (4.61 vs 4.94 mm^2^; p < 0.001), obturator internus (11.52 vs 12.98 mm^2^; p < 0.001), piriformis (10.21 vs 10.97 mm^2^; p < 0.001), and psoas (1.29 vs 1.4 mm^2^; p = 0.001) muscles; a statistically significant increase in CSA was observed for the rectus femoris (5.66 vs 5.36 mm^2^; p < 0.001), sartorius (2.98 vs 2.77 mm^2^; p < 0.001), and tensor (6.49 vs 5.58 mm^2^; p < 0.001) muscles.

**Table 4:** Measurements' mean, standard deviation and range (in brackets) and statistical differences of the cross-sectional area between the non-operated and the operated side for each approach and muscle

Muscle	Anterior		p-value	Anterolateral		p-value
	Non-operated	Operated		Non-operated	Operated	
Gluteus maximus	43.89 ± 11.36 (20.83–78.54)	44.02 ± 22.3 (21.42–91.47)	0.95	42.38 ± 13 (13.99–78.84)	40.29 ± 10.33 (25.71–65.65)	0.08
Gluteus medius	10.4 ± 2.72 (4.09–17.61)	10.46 ± 2.78 (5.07–19.58)	0.87	9.49 ± 2.68 (4.94–16.01)	9.23 ± 3.08 (4.51–17.75)	0.4
Gluteus minimus	10 ± 2.41 (5.59–18.97)	9.63 ± 2.32 (4.86–16.75)	0.009	9.51 ± 2.48 (5.17–15.91)	8.85 ± 2.6 (4.93–15.16)	0.05
Iliacus	5.35 ± 1.53 (2.31–10.83)	5.02 ± 1.48 (2.26–10.23)	< 0.001	5.19 ± 1.3 (2.21–7.46)	5.07 ± 1.02 (2.68–7.65)	0.54
Obturator internus	13.67 ± 3 (6.65–23.44)	12.02 ± 2.81 (5.23–20.07)	< 0.001	12.89 ± 2.69 (8.79–20.68)	11.68 ± 2.51 (7.24–17.44)	0.007
Obturator externus	7.64 ± 1.76 (3.84–13–84)	7.46 ± 1.64 (3.46–12.75)	0.18	8 ± 2.42 (3.44–14.98)	7.34 ± 1.85 (4.12–11.44)	0.081
Piriformis	11.1 ± 2.48 (5.33–18.78)	9.99 ± 2.66 (3.32–17.32)	< 0.001	10.89 ± 2.4 (7.16–16.85)	10.39 ± 2.69 (5.09–15.13)	0.17
Psoas	1.5 ± 0.52 (0.6–3.02)	1.38 ± 0.44 (0.54–2.94)	0.01	1.43 ± 0.48 (0.7–2.8)	1.45 ± 0.42 (0.78–2.8)	0.97
Rectus femoris	5.76 ± 1.82 (2.51–12.61)	6.02 ± 1.93 (2.52–12.63)	0.01	5.95 ± 2.12 (3.08–11.66)	6.02 ± 2.38 (3.02–12.4)	0.79
Sartorius	3.07 ± 1.03 (1.06–7.79)	3.29 ± 1.09 (1.63–9.35)	< 0.001	3.02 ± 0.86 (1.3–5.07)	3.02 ± 0.97 (1.42–5.36)	0.73
Tensor	6.07 ± 2.22 (2.6–12.59)	6.22 ± 2.01 (1.44–12.98)	0.34	5.85 ± 1.41 (3.39–8.87)	4.96 ± 1.79 (1.84–8.59)	< 0.05
Vastus lateralis	5.24 ± 1.95 (1.09–12.69)	5.4 ± 1.85 (1.31–13.67)	0.21	5.22 ± 1.9 (2.04–12.23)	5.49 ± 2.04 (1.99–10.69)	0.39

	Direct lateral		p-value	Posterior		p-value
	Non-operated	Operated		Non-operated	Operated	
Gluteus maximus	41.65 ± 10.99 (17.78–79.06)	40.62 ± 10.61 (19.74–86.41)	0.0504	44.41 ± 12.92 (21.17–96.9)	43.95 ± 11.48 (23.84–83.47)	0.73
Gluteus medius	9.9 ± 2.81 (4.27–20.64)	9.52 ± 3.11 (3.6–21.57)	0.013	10.54 ± 3.3 (4.62–23.13)	10.41 ± 3.3 (4.99–26.19)	0.81
Gluteus minimus	9.56 ± 2.26 (4.95–19.66)	8.05 ± 1.79 (3.46–12.62)	< 0.001	9.91 ± 2.47 (5.51–17.65)	9.28 ± 2.58 (4.59–19.15)	0.001
Iliacus	4.94 ± 1.46 (2.09–9.17)	4.61 ± 1.38 (2.22–9.1)	< 0.001	5.13 ± 1.64 (2.02–9.74)	4.79 ± 1.47 (2.03–10.23)	0.009
Obturator internus	12.98 ± 2.95 (6.56–20.39)	11.52 ± 2.74 (6.08–19.19)	< 0.001	12.85 ± 2.82 (7.03–22.19)	9.63 ± 2.66 (4.34–16.17)	< 0.001
Obturator externus	7.43 ± 1.83 (4.11–14.89)	7.75 ± 1.93 (3.74–13.63)	< 0.001	7.48 ± 1.78 (3.56–14.22)	7.08 ± 1.4 (4.12–11.36)	0.062
Piriformis	10.97 ± 3.08 (3.31–19.75)	10.21 ± 3.05 (3.52–20.71)	< 0.001	10.73 ± 3.25 (3.9–25.09)	8.82 ± 3.37 (1.89–23.01)	< 0.001
Psoas	1.4 ± 0.45 (0.58–2.72)	1.29 ± 0.4 (0.5–2.16)	0.001	1.42 ± 0.5 (0.49–3.49)	1.42 ± 0.58 (0.51–3.57)	0.23
Rectus femoris	5.36 ± 1.6 (2.45–9.49)	5.66 ± 1.71 (1.76–10.49)	< 0.001	5.59 ± 1.7 (2.07–9.54)	5.72 ± 1.71 (2.09–10.46)	0.12
Sartorius	2.77 ± 0.93 (0.93–5.74)	2.98 ± 0.96 (1.31–6.42)	< 0.001	2.83 ± 0.92 (1.16–4.88)	3 ± 1.01 (1.49–6.08)	< 0.001
Tensor	5.58 ± 2.16 (1.73–12.92)	6.49 ± 2.46 (1.37–14.57)	< 0.001	6.18 ± 2.89 (2.29–15.58)	5.6 ± 2.45 (2.01–17.26)	0.01
Vastus lateralis	4.83 ± 1.91 (1.19–14.33)	4.78 ± 1.61 (1.54–10.41)	0.81	4.76 ± 1.62 (1.59–12.77)	4.75 ± 1.82 (1.34–9.93)	0.45

### Fatty infiltration assessment post-operatively

**Fig. 3:**
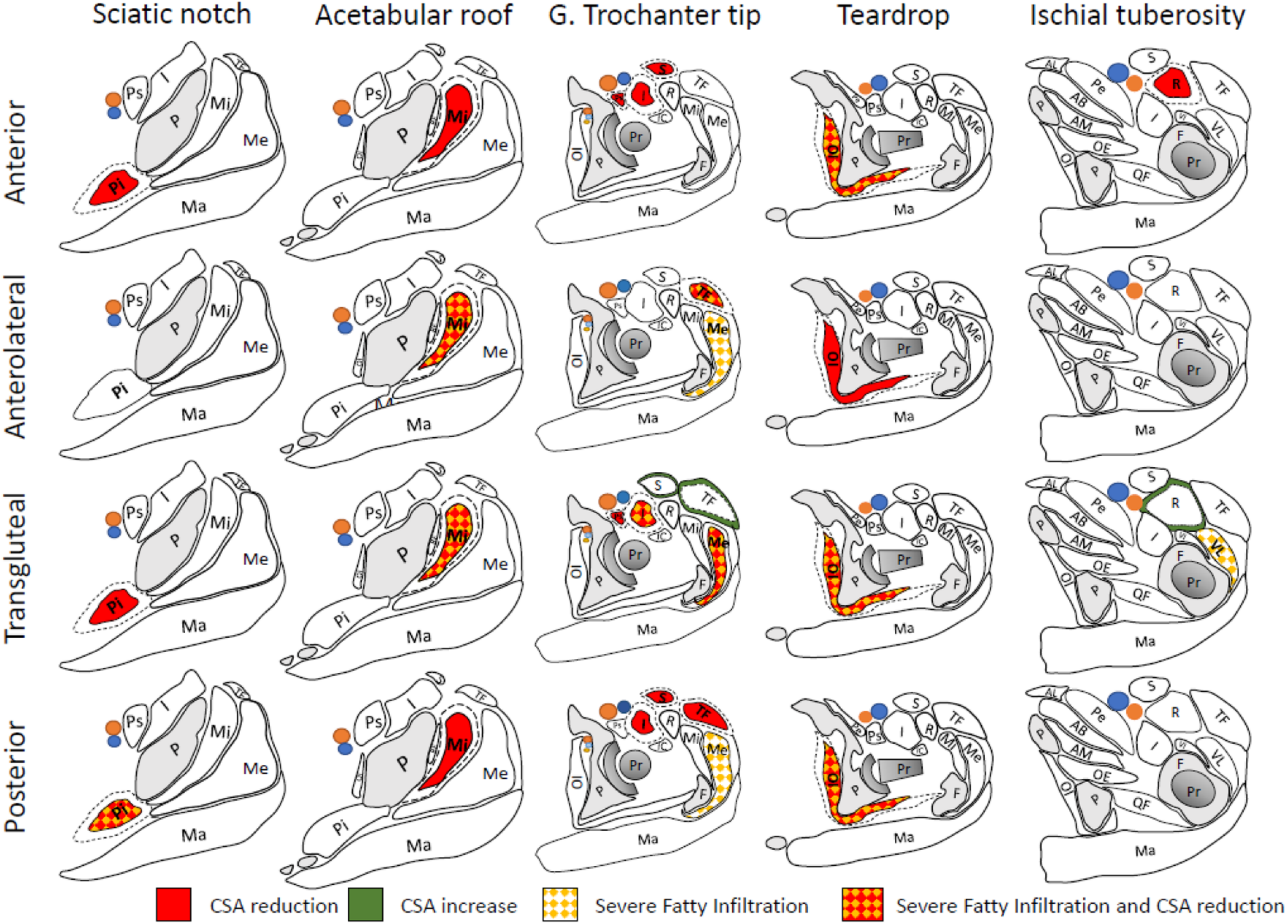
Overview of the post-operative patterns of hip muscle size reduction and fatty infiltration. Each approach is depicted at cut levels that demonstrate significant differences in cross-sectional area and fatty degeneration. See caption of Fig. 1 for anatomical features initials

Fatty infiltration was evaluated as being “non-severe” (Goutallier classification grade 0–2) and “severe” (Goutallier classification grade 3–4; Table 5; Fig. 3). For the anterior approach, we observed severe postoperative fatty infiltration of the obturator internus muscle (7.5 vs 0% severe; p < 0.001). For the anterolateral approach, we observed severe fatty infiltration of the gluteus medius (10.5 vs 0%; p = 0.041), gluteus minimus (18.4 vs 2.6%; p = 0.025), and tensor (23.7 vs 0%; p = 0.001) muscles. For the posterior approach, we observed severe fatty infiltration of the gluteus medius (3.7vs 0%; p = 0.044), obturator internus (63.2 vs 0%; p < 0.001), and piriformis (12 vs 0%; p < 0.001) muscles. Finally, for the direct lateral approach, we observed severe fatty infiltration of the gluteus medius (51.2 vs 2.5%; p < 0.001), gluteus minimus (31 vs 8.4%; p < 0.001), and vastus lateralis (4.9 vs 0%; p = 0.001) muscles.

**Table 5:** The difference in the percentage of severe fatty infiltration (Goutallier grade 3 and 4) across the observed muscles between the operated and the non-operated side for each approach

Muscle	Anterior		p-value	Anterolateral		p-value
	Non-operated	Operated		Non-operated	Operated	
Gluteus maximus	0%	0%	1	0%	0%	1
Gluteus medius	0.70%	2%	0.31	0%	10.50%	0.041
Gluteus minimus	2.10%	4.10%	0.31	2.60%	18.40%	0.025
Iliacus	0%	0%	1	0%	0%	1
Obturator Internus	0%	7.50%	< 0.001	0%	2.60%	0.317
Obturator Externus	0%	0%	1	0%	0%	1
Piriformis	0%	0.70%	0.317	0%	0%	1
Psoas	0.70%	2.10%	0.314	0%	2.60%	0.317
Rectus Femoris	0%	1.40%	0.156	0%	0%	1
Sartorius	0%	0%	1	0%	0%	1
Tensor	0%	2%	0.082	0%	23.70%	0.001
Vastus lateralis	0%	0%	1	0%	0%	1

	Direct lateral		p-value	Posterior		p-value
	Non-operated	Operated		Non-operated	Operated	
Gluteus maximus	0.50%	0.50%	1	0%	0%	1
Gluteus medius	2.50%	51.20%	< 0.001	0%	3.70%	0.044
Gluteus minimus	8.40%	31%	< 0.001	2.80%	6.50%	0.196
Iliacus	0%	0%	1	0%	0%	1
Obturator Internus	0%	1%	0.07	0%	63.20%	< 0.001
Obturator Externus	0%	0%	1	0%	2.80%	0.081
Piriformis	0%	1%	0.156	0%	12%	< 0.001
Psoas	1%	3.50%	0.092	0%	0.90%	0.317
Rectus femoris	0%	0.50%	0.317	0.90%	0.90%	1
Sartorius	0.50%	0.50%	1	0%	0.90%	0.317
Tensor	1%	0%	0.156	0%	0.9	0.317
Vastus Lateralis	0%	4.90%	0.001	0%	0%	1

## Discussion

The present study aimed at examining postoperative CT scans of a large cohort of patients who had previously undergone THA by one of four standard approaches and comparing the CSA and level of fatty infiltration of an exhaustive set of hip muscles at multiple levels to the nonoperative side. We found significant muscle size reduction and differences in fatty infiltration post-operatively across all four surgical approaches.

Overall, we observed that all approaches affected the periarticular hip musculature, with the anterolateral approach affecting the smallest number of muscles. We confirmed what has been found previously for the posterior approach, namely, it has a significant effect on the external rotators [34]. We also confirmed that the direct lateral approach has a significant effect on the abductors [13], but surprisingly, we also observed size reduction in the piriformis muscle, as well as both size reduction and fatty degeneration in the obturator internus. Given that these muscles are not supposed to be injured during the direct lateral approach, this negative impact could be a secondary effect of abductor degeneration which are some of the most important hip muscles. To our knowledge, no study has reported the above effect of the lateral approach effect on the piriformis and obturator internus to date (e.g., [2, 25, 35]). Previous research had shown that the anterior approach showed lower muscle inflammation levels than the posterior [36]. However, we found degenerative alterations in the hip external rotators (i.e., the obturator internus and the piriformis) in the anterior approach. Our finding corroborates the results of [37] and [38] showing that also minimally-invasive approaches are related to muscle degenerative patterns that likely depend on the extent of the posterolateral release. However, given the retrospective and multi-surgeon nature of our study, this is not a factor we could control for.

### Study limitations

The present study has limitations which we would like to acknowledge. The first set of limitations is related to the imaging modality used in this study. First, since the postoperative CT scans were obtained for clinical workup unrelated to the purpose of this study, there was variability between index surgery and when the CT scan was obtained. Although randomly distributed across our cohort, this temporal variability could introduce bias. We only selected CT scans that were at least 12 months post-surgery so immediate postoperative changes would not be a contributing factor. In addition, there was no preoperative scan to compare to, so changes in CSA or fatty infiltration from baseline could not be controlled for. Also, the presence of metal causes beam-hardening artifacts that can affect the interpretation of the CT scans. To address this, we excluded cases where metal artifact suppression was not included. Additionally, for CT scans that we included, we performed an inter-observer analysis to reduce potential measurement bias. Nevertheless, the CSA results for the iliacus and the psoas should be interpreted with caution due to the potential effect of metal artifact, despite using a metal suppression sequence, given their proximity to the acetabular component.

In addition to the CT-based limitations, there is a demographic difference in age between the four different groups (Table 2). Despite this difference, we did not find a substantial number of statistically significant differences between either the CSA or the fatty degeneration among the different study groups for the contralateral hip (Table 3). This would support that while there were variations in age, the relative baseline muscle health was similar. Another limitation is that muscles such as the gemelli are hard to quantify, particularly in the context of THA, and were not included in our analysis. We opted to designate certain muscles of similar location and function, such as the obturator internus, to represent muscles that were difficult to quantify. It is also important to acknowledge that this study does not include a functional evaluation, so the clinical manifestation of any such observed anatomic changes is unknown. The last main limitation is that the study does not control for factors surrounding the multiple included surgeons; namely, why a particular surgical approach was chosen by a surgeon and the surgeon’s level of comfort with the approach at the time it was used. Nevertheless, our results constitute a solid background for a future longitudinal study where these factors can be controlled.

### Anterior approach

The anterior approach was the most sparing of the abductors, consistent with previous literature [39]. We observed size reduction in the gluteus minimus, the obturator internus, psoas, and iliacus muscles. However, of these, only the obturator internus muscle showed significant fatty infiltration. This can be attributed to the release of this muscle to improve femoral exposure. The potential concerns of devascularizing the tensor muscle by ligating branches of the lateral femoral circumflex artery [40] do not seem to have a related effect on the size or fatty degeneration of the tensor postoperatively. The rectus femoris muscle and the sartorius muscle showed hypertrophy. The most likely explanation is that relative hypotrophy of these muscles, related to disuse in the setting of osteoarthritis, resolved after THA.

### Anterolateral approach

The anterolateral approach was most sparing of the external rotators. The size reduction and fatty infiltration in the gluteus minimus and tensor muscles, along with fatty infiltration of the gluteus medius (without size reduction) was a common pattern. We believe that a combination of the surgical interval and retractor placement potentially put the superior gluteal nerve, or one of its branches, at risk for injury [41]. Similar to the other approaches we studied, the obturator internus muscle demonstrated size reduction without increased fatty infiltration. We believe that the near universal finding of obturator internus changes is related to mobilization of the femur across all surgical approaches.

### Posterior approach

The posterior approach was the most harmful for the external rotators (particularly the obturator internus and piriformis muscles) [34], followed by the anterior approach, consistent with previous studies [42, 43]. In addition, the posterior approach demonstrated CSA reduction in the gluteus minimus, iliacus, sartorius and tensor muscles. The reason for this is not entirely clear, but could be related to retractor placement over the anterior lip of the acetabulum.

### Direct lateral approach

The direct lateral approach was the most harmful to the abductors, demonstrating fatty infiltration of the gluteus medius muscle in more than 50% of cases. By contrast, we observed hypertrophy of the tensor, sartorius and rectus femoris muscles. It is possible that substantial CSA reduction and fatty infiltration of the abductors, which are responsible for gait control and balance, could lead to a compensatory response from these three muscles. Previous studies have shown that a hypertrophic tensor compensates for reduced abductor size ([44, 45]). Direct incision of a part (usually the first third) of the gluteus medius, along with the detachment of the gluteus minimus, is a required part of the transgluteal approach. Our observation is that following this approach, the hip abductors do not regain their former size and/or muscle quality despite reattachment to the greater trochanter. Additionally, the vastus lateralis, which is also partially detached during this approach, demonstrates severe fatty infiltration.

## Conclusion

All four surgical approaches in our study demonstrated changes to the periarticular hip musculature. While our analysis does not distinguish a superior approach, notably, the anterior was the only approach without fatty degeneration of the gluteus medius, one of the most important muscles around the hip. Prospectively evaluating muscular changes from preop to postop, and correlating these findings with functional outcomes will help provide clarity on what surgeons can expect from each approach. Surgeons can ultimately consider these patterns of muscular changes when choosing the most appropriate surgical approach for each patient.

## Figures and Tables

**Fig. 4:**
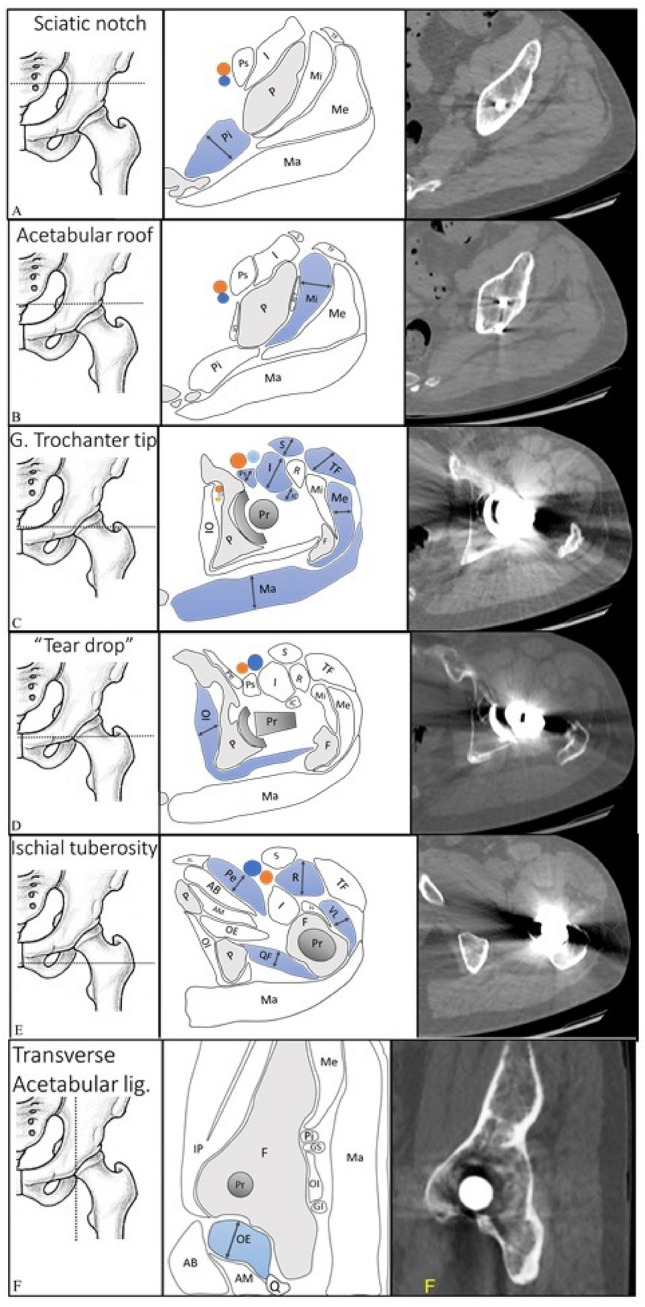
Modified protocol by Glynn et al. 2014 for the measurement of the cross-sectional area (CSA) and fatty degeneration with six cuts in the CT scan, five axial (**A**–**E**) and one sagittal (**F**). The level where each muscle was measured is colored in blue shade and is described in Table 1 in the main text. Annotation of the anatomical features illustrated: *Pelvis (P), gluteus maximus (Ma), gluteus medius (Me), gluteus minimus (Mi), tensor fasciae latae (TF), psoas (Ps), iliacus (I), sartorius (S), iliocaspuslaris (IC), rectus (R), obturator internus (OI), femur (F), prothesis (Pr), pectineus (Pe), aductor brevi (AB), aductor magnus (AM), aductor longus (AL), obturator externus (OE), gemellus superior (GS) and inferior (GI), quadratus femoris (Q), iliopsoas (IP)*

**Fig. 5:**
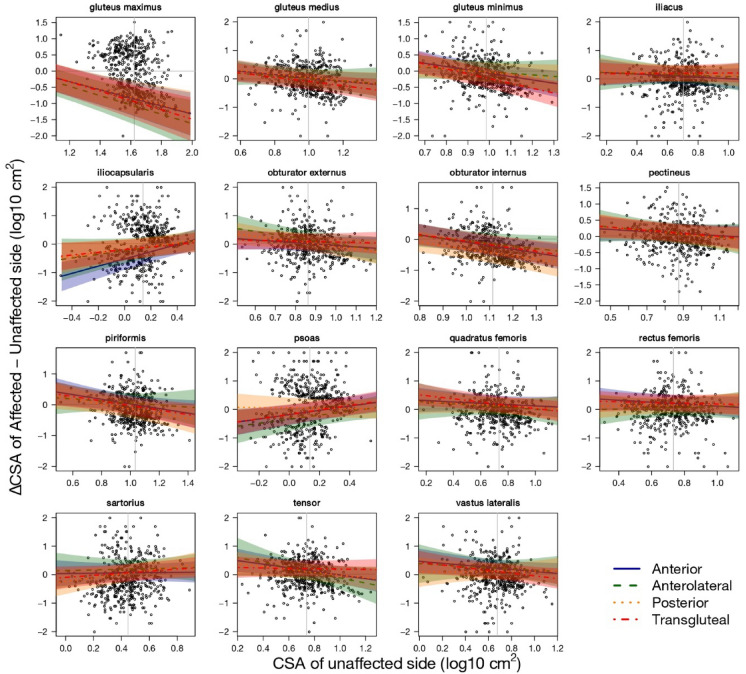
MCMCglmm predicted relationship with credible intervals (Cr. I.) between the change in CSA post-operatively (ΔCSA i.e., CSA value of the affected side—CSA value of the unaffected side) and the CSA of the unaffected side for the four types of surgical approaches (anterior, anterolateral, posterior and transgluteal) of the 15 muscles initially measured. Therefore, negative values in the y axis indicate that the operated side suffers reduction in CSA compared to the non-operated side while the grey horizontal line indicates the threshold of no difference in CSA post-operatively. The grey vertical line shows the median distribution of the CSA for the unaffected side. The values have been log10 transformed to improve model convergence and comparability among muscle CSA given the high variation of muscle size among different muscle types

**Fig. 6:**
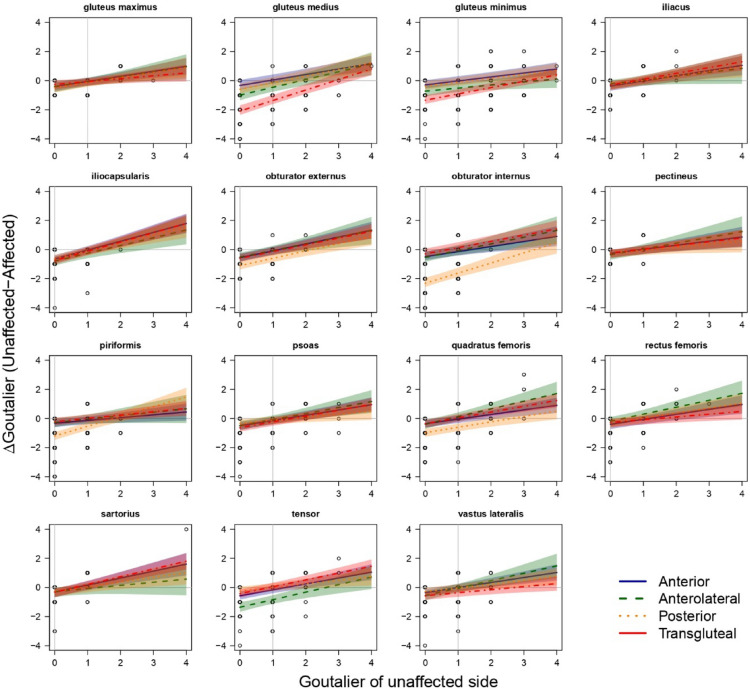
MCMCglmm predicted relationship with credible intervals (Cr. I.) between the change in fatty degeneration (Goutallier 0–4) post-operatively (ΔGoutallier i.e., Goutallier degree of fatty degeneration value of the unaffected side—Goutallier degree value of the affected side) and the Goutallier degree of fatty degeneration of the unaffected side for the four types of surgical approaches (anterior, anterolateral, posterior and transgluteal) of the 15 muscles initially measured. Therefore, negative values in the y axis indicate that the operated side suffers (further) fatty degeneration compared to the non-operated side while the grey horizontal line indicates the threshold of no difference in fatty degeneration post-operatively. The grey vertical line shows the median distribution of the Goutallier for the unaffected side

## Data Availability

The study has been approved by the Swiss Ethics and Research Committee of the Canton of Vaud (local Institute for Research in Biomedicine, IRB; CER-VD 2021-00577) and complies with the ethical standards of the 1964 Declaration of Helsinki and the relevant regulations of the US Health Insurance Portability and Accountability Act (HIPAA). The authors used patient data only that support the findings of this study, but are not openly available due to reasons of sensitivity and are available from the corresponding author upon reasonable request. Data are located in controlled access data storage at HFR Cantonal Hospital server.

## Appendix A

See Figs. 4,5 and 6

This supplemental material is included with our main report to provide a more in-depth analysis of our collected data.

## Statistical analysis

### Quantification of the effect of approach on muscle size and fatty infiltration with Bayesian statistics

For a more in-depth understanding of the effect of each approach on muscle size and fatty infiltration, we fitted a Marcov Chain Monte Carlo generalized linear mixed-effects model (MCMCglmm [33],) in five parallel chains, one model for CSA and one for fatty infiltration, this time keeping all degrees of Goutallier classification (i.e., 0–4). We used the nonoperative side measurement as a fixed effect, and the patient, muscle, and the interaction of the random slope of the nonoperative side muscle size with the type of approach per muscle as random effects. Using the nonoperative side as a reference, the response variable of the model was the difference in CSA or degree of fatty infiltration of the operative side from the nonoperative side. This analysis (run in R statistical software [46]) assessed the effect of each approach on every muscle considering the muscle size, resulting in a detailed quantitative comparison of each surgical approach’s performance in regard to all 15 muscles initially measured.

## Results

### CSA post-operatively

The comparative quantification of CSA performance of each surgical approach using MCMCglmm is shown in detail in Figure A.2. Muscle size tended to reduce post-operatively for all muscles and approaches at different magnitudes (based on the random slope/rate of change of the response variable) of the MCMCglmm except for the Rectus Femoris (Anterolateral approach) and the Sartorius (Posterior and transgluteal approaches). However, for the same approaches, when the above muscles had a smaller initial size, the affect was more negative. Overall, the anterolateral approach had the least negative impact in CSA with increasing muscle size across all muscles. This was followed by the anterior approach, the transgluteal approach, and the posterior approach, respectively.

### Fatty infiltration assessment post-operatively

The comparative quantitative performance of each surgical approach per muscle in fatty infiltration (classes 0–4) based on MCMCglmm showed that across all approaches and muscles, the fatty degeneration of the operative side was increased, especially for muscles with low non-operative side fatty degeneration (see B0 in Figure A.2). Interestingly, the opposite pattern was found with higher non-operative side fatty degeneration. For the abductors in particular, the transgluteal approach increased the fatty degeneration of the operated side the most, followed by the anterolateral approach. The anterior approach performed better than other approaches for fatty infiltration of the abductors. The transgluteal and anterolateral approaches performed better than both anterior and posterior approaches for fatty infiltration of the external rotators. The posterior approach demonstrated the worst fatty infiltration for the external rotators.

## References

[1] Angerame MR, Dennis DA (2018) Surgical approaches for total hip arthroplasty. Ann Joint 3:43–43

[2] Caton J, Prudhon JL (2011) Over 25 years survival after Charnley’s total hip arthroplasty. Int Orthop 35:185–188. 10.1007/s00264-010-1197-z 21249358 10.1007/s00264-010-1197-z PMC3032109

[3] Learmonth ID, Young C, Rorabeck C (2007) The operation of the century: total hip replacement. Lancet 370:1508–1519. 10.1016/S0140-6736(07)60457-7 17964352 10.1016/S0140-6736(07)60457-7

[4] Beck M, Brand C, Christen B, Zdravkovic V (2023) Swiss national Hip & Knee joint Registry—Report 2023—Annual report of the SIRIS Registry Hip & Knee, 2012-2022, p 39

[5] Galakatos GR (2018) Direct Anterior Total Hip Arthroplasty. J Mo State Med Assoc 115(6):537–541 PMC6312152 30643349

[6] Judet J, Judet R (1950) The use of an artificial femoral head for arthroplasty of the hip joint. J Bone Joint Surg Br. 10.1302/0301-620X.32B2.166 15422013 10.1302/0301-620X.32B2.166

[7] Rachbauer F, Kain MSH, Leunig M (2009) The history of the anterior approach to the Hip. Orthop Clin North Am 40:311–320. 10.1016/j.ocl.2009.02.007 19576398 10.1016/j.ocl.2009.02.007

[8] Hansen BJ, Hallows RK, Kelley SS (2011) The Rottinger approach for total hip arthroplasty: technique and review of the literature. Curr Rev Musculoskelet Med 4:132–138. 10.1007/s12178-011-9093-8 21826433 10.1007/s12178-011-9093-8 PMC3261250

[9] Moore AT (1957) The self-locking metal hip prosthesis. J Bone Joint Surg 39:811–827. 10.2106/00004623-195739040-00005 13438939

[10] Bauer R, Kerschbaumer F, Poisel S, Oberthaler W (1979) The transgluteal approach to the hip joint. Arch Orthop Trauma Surg 95:47–49. 10.1007/BF00379169 526126 10.1007/BF00379169

[11] Hardinge K (1982) The direct lateral approach to the hip. J Bone Joint Surg Ser B 64:17–19. 10.1302/0301-620x.64b1.7068713 10.1302/0301-620X.64B1.7068713 7068713

[12] Lepri AC, Villano M, Matassi F, Carulli C, Innocenti M, Civinini R (2020) “Anterolateral” approach to the hip: a systematic review of the correct definition of terms. Hip Int 30:13–19. 10.1177/1120700020966800 33267690 10.1177/1120700020966800

[13] Bremer AK, Kalberer F, Pfirrmann CWA, Dora C (2011) Soft-tissue changes in hip abductor muscles and tendons after total hip replacement: comparison between the direct anterior and the transgluteal approaches. J Bone Joint Surg Ser B. 10.1302/0301-620X.93B7.25058 10.1302/0301-620X.93B7.25058 21705558

[14] Müller M, Schwachmeyer V, Tohtz S, Taylor WR, Duda GN, Perka C et al (2012) The direct lateral approach: impact on gait patterns, foot progression angle and pain in comparison with a minimally invasive anterolateral approach. Arch Orthop Trauma Surg 132:725–731. 10.1007/s00402-012-1467-x 22294091 10.1007/s00402-012-1467-x

[15] Müller M, Tohtz S, Dewey M, Springer I, Perka C (2010) Evidence of reduced muscle trauma through a minimally invasive anterolateral approach by means of MRI. Clin Orthop Relat Res 468:3192–3200. 10.1007/s11999-010-1378-5 20458641 10.1007/s11999-010-1378-5 PMC2974868

[16] Amlie E, Havelin LI, Furnes O, Baste V, Nordsletten L, Hovik O et al (2014) Worse patient-reported outcome after lateral approach than after anterior and posterolateral approach in primary hip arthroplasty: a cross-sectional questionnaire study of 1476 patients 1–3 years after surgery. Acta Orthop 85:463–469. 10.3109/17453674.2014.934183 24954494 10.3109/17453674.2014.934183 PMC4164862

[17] Kwon MS, Kuskowski M, Mulhall KJ, Macaulay W, Brown TE, Saleh KJ (2006) Does surgical approach affect total hip arthroplasty dislocation rates? Clin Orthop Relat Res. 10.1097/01.blo.0000218746.84494.df 16741471 10.1097/01.blo.0000218746.84494.df

[18] Pellicci PM, Bostrom M, Poss R (1998) Posterior approach to total hip replacement using enhanced posterior soft tissue repair. Clin Orthop Relat Res. 10.1097/00003086-199810000-00023 9917607 10.1097/00003086-199810000-00023

[19] Hasija R, Kelly JJ, Shah NV, Newman JM, Chan JJ, Robinson J et al (2018) Nerve injuries associated with total hip arthroplasty. J Clin Orthop Trauma 9:81–86. 10.1016/j.jcot.2017.10.011 29628688 10.1016/j.jcot.2017.10.011 PMC5884042

[20] Mardones R, Pagnano MW, Nemanich JP, Trousdale RT (2005) The frank stinchfield award: muscle damage after total hip arthroplasty done with the two-incision and mini-posterior techniques. Clin Orthop Relat Res 441:63–67. 10.1097/01.blo.0000194727.55372.04 16330985 10.1097/01.blo.0000194727.55372.04

[21] Meneghini RM, Pagnano MW, Trousdale RT, Hozack WJ (2006) Muscle damage during MIS total hip arthroplasty: smith-peterson versus posterior approach. Clin Orthop Relat Res 453:293–298. 10.1097/01.blo.0000238859.46615.34 17006366 10.1097/01.blo.0000238859.46615.34

[22] Parratte S, Pagnano MW (2008) Muscle damage during minimally invasive total hip arthroplasty: cadaver-based evidence that it is significant. Instr Course Lect 57:231–234 18399584

[23] Kawano T, Nankaku M, Murao M, Hamada R, Goto K, Kuroda Y et al (2022) Recovery of muscle atrophy and fatty infiltration in patients with acetabular dysplasia after total hip arthroplasty. J Am Acad Orthop Surg 30:e317–e326. 10.5435/JAAOS-D-21-00156 34910715 10.5435/JAAOS-D-21-00156

[24] Lüdemann M, Kreutner J, Haddad D, Kenn W, Rudert M, Nöth U (2012) MRT-basierte messung des muskelschadens nach minimal-invasiver hüftprothesenimplantation. Orthopäde 41:346–353. 10.1007/s00132-011-1889-0 22552541 10.1007/s00132-011-1889-0

[25] Pfirrmann CWA, Notzli HP, Dora C, Hodler J, Zanetti M (2005) Abductor tendons and muscles assessed at MR imaging after total hip arthroplasty in asymptomatic and symptomatic patients. Radiology 235:969–976. 10.1148/radiol.2353040403 15860673 10.1148/radiol.2353040403

[26] Kovalak E, Özdemir H, Ermutlu C, Obut A (2018) Assessment of hip abductors by MRI after total hip arthroplasty and effect of fatty atrophy on functional outcome. Acta Orthop Traumatol Turc 52:196–200. 10.1016/j.aott.2017.10.005 29478777 10.1016/j.aott.2017.10.005 PMC6136339

[27] Glynn AA, Barattiero FY, Albers CE, Hanke MS, Steppacher SD, Tannast M (2014) Surgical hip dislocation does not result in atrophy or fatty infiltration of periarticular hip muscles. J Hip Preserv Surg 1:82–95. 10.1093/jhps/hnu008 27011807 10.1093/jhps/hnu008 PMC4765291

[28] Bogunovic L, Lee SX, Haro MS, Frank JM, Mather RC, Bush-Joseph CA et al (2015) Application of the goutallier/fuchs rotator cuff classification to the evaluation of hip abductor tendon tears and the clinical correlation with outcome after repair. Arthrosc J Arthrosc Relat Surg 31:2145–2151. 10.1016/j.arthro.2015.04.101 10.1016/j.arthro.2015.04.101 26188781

[29] Goutallier D, Postel JM, Bernageau J, Lavau L, Voisin MC (1994) Fatty muscle degeneration in cuff ruptures: Pre- and postoperative evaluation by CT scan. Clin Orthop Relat Res. 10.1097/00003086-199407000-00014 8020238

[30] Kruskal WH, Wallis WA (1952) Use of ranks in one-criterion variance analysis. J Am Stat Assoc 47:583–621. 10.1080/01621459.1952.10483441

[31] Pearson KX (1900) On the criterion that a given system of deviations from the probable in the case of a correlated system of variables is such that it can be reasonably supposed to have arisen from random sampling. Lond Edinb Dublin Philos Mag J Sci 50:157–175. 10.1080/14786440009463897

[32] Wilcoxon F (1945) Individual comparisons by ranking methods. Bull Biom 1:80–83

[33] Hadfield JD (2015) Increasing the efficiency of MCMC for hierarchical phylogenetic models of categorical traits using reduced mixed models. Method Ecol Evol 6:706–714. 10.1111/2041-210X.12354

[34] Wang T, Shao L, Xu W, Li F, Huang W (2019) Surgical injury and repair of hip external rotators in THA via posterior approach: a three-dimensional MRI-evident quantitative prospective study. BMC Musculoskelet Disord 20:22. 10.1186/s12891-018-2367-1 30642331 10.1186/s12891-018-2367-1 PMC6332581

[35] Agten CA, Sutter R, Dora C, Pfirrmann CWA (2017) MR imaging of soft tissue alterations after total hip arthroplasty: comparison of classic surgical approaches. Eur Radiol 27:1312–1321. 10.1007/s00330-016-4455-7 27342822 10.1007/s00330-016-4455-7

[36] Bergin PF, Doppelt JD, Kephart CJ, Benke MT, Graeter JH, Holmes AS et al (2011) Comparison of minimally invasive direct anterior versus posterior total hip arthroplasty based on inflammation and muscle damage markers. J Bone Joint Surg 93:1392–1398. 10.2106/JBJS.J.00557 21915544 10.2106/JBJS.J.00557 PMC3143583

[37] Eilander W, Van Der Velden E, Van Harten M, Van Kampen P, Hogervorst T (2023) The short external rotators in the anterior approach hip arthroplasty: do the tendons heal or not? A prospective MRI study. HIP Int 33:819–827. 10.1177/11207000221107551 35765171 10.1177/11207000221107551

[38] Rykov K, Meys TWGM, Knobben BAS, Sietsma MS, Reininga IHF, Ten Have BLEF (2021) MRI assessment of muscle damage after the posterolateral versus direct anterior approach for THA (Polada trial). A randomized controlled trial. J Arthroplast. 10.1016/j.arth.2021.05.009 10.1016/j.arth.2021.05.009 34116911

[39] Oda S, Hisatome T, Cho E, Fujimaki H, Nakanishi K (2022) MRI findings of muscle damage after total hip arthroplasty using the complete muscle preserving anterolateral supine approach. Medicina 58:713. 10.3390/medicina58060713 35743976 10.3390/medicina58060713 PMC9228776

[40] Kalhor M, Gharehdaghi J, Leunig M, Ganz R (2022) Lateral femoral circumflex artery contribution to the articular and periarticular hip circulation: relevance to the anterior hip approach—a cadaveric study. Eur J Orthop Surg Traumatol. 10.1007/s00590-022-03310-2 35727417 10.1007/s00590-022-03310-2

[41] Takada R, Jinno T, Miyatake K, Hirao M, Yoshii T, Okawa A (2021) Incidence of tensor fascia lata muscle atrophy after using the modified Watson-Jones anterolateral approach in total hip arthroplasty. Eur J Orthop Surg Traumatol 31:533–540. 10.1007/s00590-020-02806-z 33040212 10.1007/s00590-020-02806-z

[42] Eilander W, van der Velden E, van Harten M, van Kampen P, Hogervorst T (2022) The short external rotators in the anterior approach hip arthroplasty: do the tendons heal or not? A prospective MRI study. HIP Int. 10.1177/11207000221107551 35765171 10.1177/11207000221107551

[43] Robinson J, Bas M, Deyer T, Cooper HJ, Hepinstall M, Ranawat A et al (2022) Muscle recovery after total hip arthroplasty: prospective MRI comparison of anterior and posterior approaches. HIP Int. 10.1177/11207000221114456 36192819 10.1177/11207000221114456

[44] Rodríguez-Roiz JM, Bori G, Tomas X, Fernández-Valencia JA, García-Díez AI, Pomés J et al (2017) Hypertrophy of the tensor fascia lata muscle as a complication of total hip arthroplasty. Eur J Orthop Surg Traumatol 27:255–259. 10.1007/s00590-016-1854-z 27644425 10.1007/s00590-016-1854-z

[45] Sutter R, Kalberer F, Binkert CA, Graf N, Pfirrmann CWA, Gutzeit A (2013) Abductor tendon tears are associated with hypertrophy of the tensor fasciae latae muscle. Skelet Radiol 42:627–633. 10.1007/s00256-012-1514-2 10.1007/s00256-012-1514-2 22940837

[46] R Core Team (2018). R: A language and environment for statistical computing (version 3.4.0). R foundation for statistical computing. https://www.R-project.org/ n.d.

